# Effects of propofol/remifentanil-based total intravenous anesthesia versus sevoflurane-based inhalational anesthesia on the release of VEGF-C and TGF-β and prognosis after breast cancer surgery: a prospective, randomized and controlled study

**DOI:** 10.1186/s12871-018-0588-3

**Published:** 2018-09-22

**Authors:** Tao Yan, Guo-Hua Zhang, Bao-Na Wang, Li Sun, Hui Zheng

**Affiliations:** Department of Anesthesiology, National Cancer Center/National Clinical Research Center for Cancer/Cancer Hospital, Chinese Academy of Medical Sciences and Peking Union Medical College, No.17 Pan-Jia-Yuan nanli Road, Chaoyang District, Beijing, 100021 China

**Keywords:** Breast cancer, Angiogenesis, Propofol, Sevoflurane, Total intravenous anesthesia

## Abstract

**Background:**

Vascular endothelial growth factor (VEGF) and transforming growth factor-β (TGF-β) have been involved in tumor growth and metastasis. Sevoflurane may promote angiogenesis, whereas propofol can present an anti-angiogenic effect. In this study, we compared the effects of propofol/remifentanil-based total intravenous anesthesia (TIVA) and sevoflurane-based inhalational anesthesia on the release of VEGF-C and TGF-β, as well as recurrence- free survival (RFS) rates in the patients undergoing breast cancer surgery.

**Methods:**

Eighty female patients undergoing breast cancer resection were enrolled and randomized to receive either sevoflurane-based inhalational anesthesia (SEV group) or propofol/remifentanil-based TIVA (TIVA group). The serum concentrations of VEGF-C and TGF-β before and 24 h after surgery were measured and RFS rates over a two-year follow-up were analyzed in both groups. The postoperative pain scores assessed using a visual analogue scale (VAS) and the use of perioperative opioids were also evaluated.

**Results:**

Although VAS scores at 2 h and 24 h after surgery were comparable between the two groups, there were more patients receiving postoperative fentanyl in the TIVA group (16[40%]) compared with the SEV group (6[15%], *p* = 0.023). VEGF-C serum concentrations increased after surgery from 105 (87–193) pg/ml to174 (111–281) pg/ml in the SEV group (*P* = 0.009), but remained almost unchanged in the TIVA group with 134 (80–205) pg/ml vs.140(92–250) pg/ml(*P* = 0.402). The preoperative to postoperative change for VEGF-C of the SEV group (50 pg/ml) was significantly higher than that of the TIVA group (12 pg/ml) with a difference of 46 (− 11–113) pg/ml (*P* = 0.008). There were also no significant differences in the preoperative and postoperative TGF-β concentrations between the two groups. The two-year RFS rates were 78% and 95% in the SEV and TIVA groups (*P* = 0.221), respectively.

**Conclusion:**

In comparison with sevoflurane-based inhalational anesthesia, propofol/remifentanil -based total intravenous anesthesia can effectively inhibit the release of VEGF-C induced by breast surgery, but didn’t seem to be beneficial in the short-term recurrence rate of breast cancer.

**Trial registration:**

Chictr.org.cn ChiCTR1800017910. Retrospectively Registered (Date of registration: August 20, 2018).

## Background

Breast cancer is one of the most common malignant tumors and remains a leading cause of cancer mortality among women [1]. Surgical excision is the principle treatment of such solid tumors. However, it has been widely recognized that both surgical manipulation and anesthetic techniques can alter immunologic function to potentially influence cancer recurrence and metastasis [2, 3]. Sevoflurane can suppress cell-mediated immunity (CMI) and induce T lymphocyte apoptosis, and it has also been shown to increase proliferation, mitigation and invasion of breast cancer cells [4–6]. In contrast, propofol predominantly exhibited anti-tumor effects and was conducive to maintain anti-tumor immunity through promoting the activation and differentiation of T helper lymphocytes [7–9]. The effects of opioids on tumor growth and metastasis appeared to be more complex and controversial, depending on drug concentration or exposure duration, or even type of cancer [10–12]. Furthermore, perioperative administration of opioids attenuated surgery-induced stress response, thereby modulating tumor promoting effects of surgery [13, 14].

The previous studies suggested that propofol/paravertebral anesthesia was associated with unchanged serum concentration of vascular endothelial growth factor (VEGF) after breast cancer surgery and greater inhibition of tumor cell proliferation compared with sevoflurane/opioids anesthesia [2, 15]. However, paravertebral block is usually performed under ultrasound guidance which may not be available in some hospitals, and thus limits the favorable results to be generalized to most of breast cancer patients. Conventional method of propofol based anesthesia typically consists of remifentanil and fentanyl, which could further confound the effects of propofol on immune function. Nonetheless, propofol/opioids-based total intravenous anesthesia (TIVA) has been proven to be associated with reduced risk of recurrence after modified radical mastectomy (MRM) in a retrospective study, but where perioperative angiogenic factors were not measured [3]. Moreover, whether propofol/opioids-based TIVA could still render anti-tumor properties for the patients receiving breast conserving surgery (BCS), a surgical procedure inducing less stress response than MRM, has not been evaluated.

Therefore, we conducted the present study to investigate the influences of two commonly used anesthetic methods, propofol/opioids-based TIVA and sevoflurane-based inhalational anesthesia, on the release of angiogenic factors including VEGF and transforming growth factor-β(TGF-β) which play a crucial role in tumor progression [16–18], and on the recurrence-free survival (RFS) and overall survival (OS) rates in the patients undergoing MRM or BCS.

## Methods

### Study design

This study was a prospective, controlled, parallel-group clinical trial with equal randomization and was performed at Cancer Hospital of Chinese Academy of Medical Sciences. Ethic approval was obtained from Ethic Committee of Cancer Hospital (approval number: NCC2013YZ-06). The study was registered at Chictr.org.cn registry system on 20 August 2018 (ChiCTR1800017910). The enrollment started from January 2016 and ended at August 2016.The follow-up was completed at June 2018.

### Participants

After taking written informed consent, adult female patients aged 18 to 80 years, ASA physical status Iand II, undergoing MRM or BCS for confirmed breast cancer were enrolled in the study. MRM was defined as mastectomy and axillary clearance. BCS was referred to wide local excision of breast tissue and axillary nodes sampling/clearance. Exclusion criteria included age younger than 18 or older than 80 years old; previous breast cancer surgery (except tumor biopsy); bilateral breast surgery; preoperative chemotherapy; contraindication to use of sevoflurane, propofol and nonsteroidal anti-inflammatory drugs (NSAIDs).

### Randomization

The patients were randomly assigned to receive propofol/remifentanil -based TIVA (TIVA group) or sevoflurane- based inhalational anesthesia (SEV group). Randomization was done using a sealed envelope system. A physician (Dr. Liu) not involved in the study randomly inserted 50 of each two anesthetic designations to 100 sequentially numbered envelopes. The allocation sequence was generated using a random number generator. The envelop was opened before anesthetic induction by the investigators to determine which anesthetic technique was going to be performed. The non-operational investigator (Dr. BNW) was responsible for taking outcome measurements. Individuals who conducted laboratory assays were kept blinded to allocation.

### Interventions

In the operating room, hemodynamic and bispectral index (BIS) monitoring were applied. General anesthesia was induced with fentanyl 2-3μg/kg, propofol 1-2 mg/kg and rocuronium 0.6 mg/kg in both groups. After insertion of a laryngeal mark airway, the patients were mechanically ventilated to maintain the end tidal carbon dioxide concentration at 35-45 mmHg with a fresh gas flow of 2 L/min oxygen. Anesthesia was maintained with constant infusion of propofol at a rate of 3-6 mg/kg/h and remifentanil at a rate of 0.1–0.2μg/kg/min in the TIVA group or 1.5–2% sevoflurane in the SEV group to maintain BIS values of 40–60. Fentanyl 1μg/kg was added intraoperatively as required. At the end of surgery all patients received 50 mg of flurbiprofen (a kind of NSAIDs). Postoperative analgesia consisted of flurbiprofen 50 mg as the initial choice, and then fentanyl 1μg/kg, if necessary, which were given when the patients complained of pain in the post-anesthesia care unit and ward. All patients received standard postoperative therapies according to the pathological characteristics.

### Outcomes

The primary outcome was the preoperative to postoperative change of VEGF-C concentrations and used to power the study. Venous blood was sampled using serum separator tubes (BD, Franklin Lakes, NJ) before and 24 h after surgery. Samples were centrifuged at 3000×g for 5 min at 4-degree Celsius (°C) and the supernatants were transferred into secondary centrifuge tubes, followed by centrifuging at 12,000×g for 5 min at 4 °C to remove the remaining cells. The resultant serum was stored at − 80 °C for further analysis. Serum VEGF-C and TGF-β concentrations were analyzed using the Enzyme-linked immunosorbent assay (ELISA)kits (CUSABIO Life Science, Wuhan, China) according to the manufacturer’s instructions. The level of each angiogenic factor was determined by calculating its optical density at 450 nm (nm) using a spectrophotometer. The minimal detectable dose limit for each kit was as follows: VEGF-C, 3 pg/ml; TGF-β, 6 pg/ml.

Secondary outcomes included changes in TGF-β concentrations, pain assessment scores, the number of patients receiving intraoperative fentanyl, and the number of those being given postoperative flurbiprofen and/or fentanyl for pain relief, as well as RFS and OS rates. Postoperative pain was assessed using a 10-cm visual analogue scale (VAS) at 2 h and 24 h after surgery. RFS and OS rates were estimated by a two-year follow-up. RFS was defined as the time from the date of surgery till disease relapse confirmed by clinical evidence and radiological examination. OS was defined as the time from the date of surgery till death or last follow-up. The last date of follow-up was June 1th, 2018 and patients alive on the last follow-up date were considered censored.

Additional perioperative data collected were patients’ demographic data, anesthetic and surgical factors such as tumor size, pathological grade, estrogen receptor (ER) status, progesterone receptor (PR) status, epidermal growth factor receptor2 (HER2) expression, tumor staging.

### Sample size and statistical analysis

The sample size was calculated based on the primary outcome. According to the previous study [2], it was assumed that VEGF serum concentration would be increased by 50% after surgery in the SEV group and by 10% in the TIVA group, which would show a significant change between the two groups. Our preliminary study showed a mean (SD)VEGF increase of 80(60) pg/ml after surgery in the SEV group, achieving a power of 0.8 at a α level of 0.05, there would be at least fifteen patients in each group to detect a significant difference. However, it should be noted that there were two types of surgical procedures in each group, MRM and BCS, which could produce different intensity of stress response, hence the release of angiogenic factors. We also wanted to compare the changes of VEGF concentrations among the patients receiving MRM between the two groups, and also for those undergoing BCS to reduce the surgical associated interference as much as possible. Therefore, considering some participants would be lost, total eighty patients should be recruited to guarantee at least fifteen patients receiving either MRM or BCS in each group.

Data were analyzed using SPSS 25.0 for windows (SPSS, Inc., Chicago, IL, USA). An independent *t* test and a Mann–Whitney U test were used for parametric and nonparametric data analysis, respectively. Categorical data were assessed by Fisher exact test. Recurrence-free survival and overall survival were estimated by a Kaplan Meier log-rank test. Data are expressed as median (25–75% interquartile range) or mean (SD). A *p* value of less than 0.05 was considered statistically significant.

## Results

### Participants

**Fig. 1:**
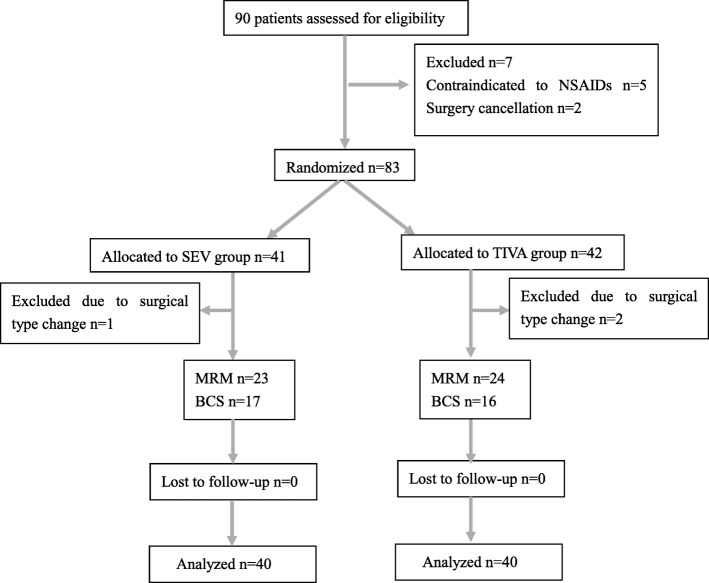
CONSORT flow diagram of participants allocation. SEV sevoflurane, TIVA total intravenous anesthesia, MRM modified radical mastectomy, BCS breast conserving surgery

Participants who were randomly allocated to each of the anesthetic groups and analyzed for the primary outcome are shown in the CONSORT flow diagram (see Fig. 1.) There were total 90 patients assessed for eligibility. Ten patients were excluded from this study with five contraindicated to NSAIDs, two not receiving surgery, three experiencing change of surgical types. Finally, there were 80 patients enrolled in the study, 23 cases for MRM and 17 for BCS in the SEV group, 24 cases for MRM and 16 for BCS in the TIVA group. There were no significant differences in terms of patient characteristics and surgical factors between the two groups, as shown in Table 1.

**Table 1:** Patient characteristics and surgical factors

	Group SEV (*n* = 40)	Group TIVA (*n* = 40)	*p*-value
Age (years)	49.5 (9.5)	48.7 (10.6)	0.704
Height (cm)	161.6 (4.6)	161.2 (5.5)	0.663
Weight (kg)	58.2 (7.2)	57.0 (6.3)	0.630
ASA grade			0.446
I	31 (77.5)	28 (70)	
II	9 (22.5)	12 (30)	
Tumor size (mm)	2.1 (0.9)	2.2 (1.0)	0.626
Histological Grade			0.644
I	7 (17.5)	8 (20.0)	
II	17 (42.5)	20 (50.0)	
III	16 (40.0)	12 (30.0)	
Carcinoma cell embolus	11 (27.5)	17 (42.5)	0.160
Nerve invasion	5 (12.5)	5 (12.5)	1.000
Positive receptors			
Estrogen	26 (65.0)	32 (80.0)	0.133
Progesterone	22 (55.0)	27 (67.5)	0.251
HER2 expression	15 (37.5)	12 (30.8)	0.528
TNM Staging			0.995
Tis	1 (2.5)	1 (2.5)	
Stage I	12 (30.0)	13 (32.5)	
Stage II	19 (47.5)	18 (45.0)	
Stage III	8 (20.0)	8 (20.0)	
Duration of anesthesia (min)	79.0 (30.5)	79.4 (26.3)	0.592
Surgical approach			0.820
MRM	23 (57.5)	24 (60.0)	
BCS	17 (42.5)	16 (40.0)	

Values are mean (SD) or number of patients (n, %)

*Abbreviation*: *SEV* sevoflurane, *TIVA* total intravenous anesthesia, *ASA* American Society of Anesthesiologists, *TNM* Tumor-Node-Metastasis, *Tis* tumor in situ, *HER2* human epidermal growth factor receptor 2, *MRM* modified radical mastectomy, *BCS* breast conserving surgery

### Postoperative pain assessment

Although VAS scores at 2 h (3 [2, 3] vs. 3 [2, 3], *P* = 0.697) and 24 h (2 [1, 2] vs. 2 [1–3], *P* = 0.098) after surgery were comparable between the two groups, there were more patients receiving postoperative fentanyl in the TIVA group (16[40%]) compared with the SEV group (6[15%], *P* = 0.023) (see Table 2).

**Table 2:** Pain and analgesia data

	Group SEV (*n* = 40)	Group TIVA (n = 40)	*P*-value
VAS score			
2 h after surgery	3(2–3)	3(2–3)	0.697
24 h after surgery	2(1–2)	2(1–3)	0.098
Intraoperative fentanyl	20(50)	18(45)	0.823
Postoperative analgesia			
fentanyl	6(15)	16(40)	0.023
flurbiprofen	28(70)	30(75)	0.803

Values are median (25–75% interquartile range) or number of patients (n, %)

*Abbreviation*: *SEV* sevoflurane, *TIVA* total intravenous anesthesia, *VAS* visual analogue scale

### Serum levels of angiogenic factors before and after surgery

**Fig. 2:**
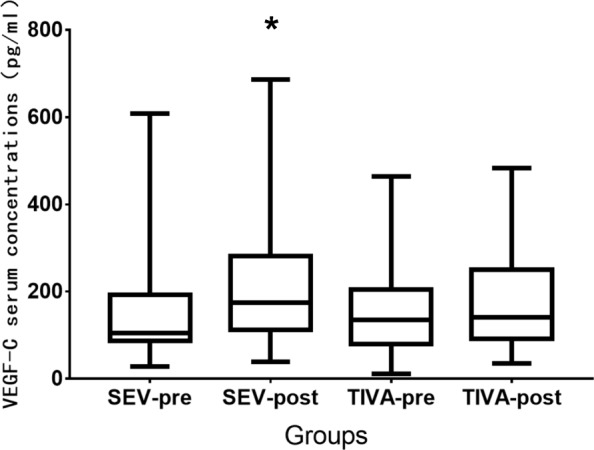
Median preoperative and postoperative VEGF-C concentrations in both groups. *P = 0.009,higher postoperative values versus preoperative values in the SEV group. SEV-pre preoperative values in the SEV group, SEV-post postoperative values in the SEV group, TIVA-pre preoperative values in the SEV group, TIVA-post postoperative values in the TIVA group. Horizontal line denotes median values, box borders refer to interquartile range, whiskers indicate range of values

VEGF-C serum concentrations increased after surgery from 105 (87–193) pg/ml to174 (111–281) pg/ml in the SEV group (*P* = 0.009), but remained almost unchanged in the TIVA group with 134 (80–205) pg/ml versus 140(92–250) pg/ml (*P* = 0.402). Both preoperative and postoperative VEGF-C concentrations showed no significant differences when comparing the two groups, however, the median preoperative to postoperative change score for VEGF-C of the SEV group (50 pg/ml) was significantly higher than that of the TIVA group (12 pg/ml) with a difference (*P* = 0.008) of 46 (− 11–113) pg/ml (see Table 3 and Fig. 2).

**Table 3:** Comparisons of angiogenic factors concentrations (pg/ml) between the two groups

	Group SEV (*n* = 40)	GroupTIVA (*n* = 40)	*P*-value
Preoperative values			
VEGF-C	105 (87–193)	134 (80–205)	0.729
TGF-β	197(131–318)	198(100–304)	0.721
Postoperative values			
VEGF-C	174 (111–281)	140(92–250)	0.177
TGF-β	211(109–308)	176(116–361)	0.794
Pre-post changes^a^			
VEGF-C	50(21–108)	12(−8–52)	0.008
TGF-β	3(−30–47)	13(−17–51)	0.582

Values are median (25–75% interquartile range)

*Abbreviation*: *SEV* sevoflurane, *TIVA* total intravenous anesthesia, *VAS* visual analogue scale, *VEGF* vascular endothelial growth factor, *TGF* transforming growth factor

^a^Pre-post changes: postoperative values minus preoperative values

**Fig. 3:**
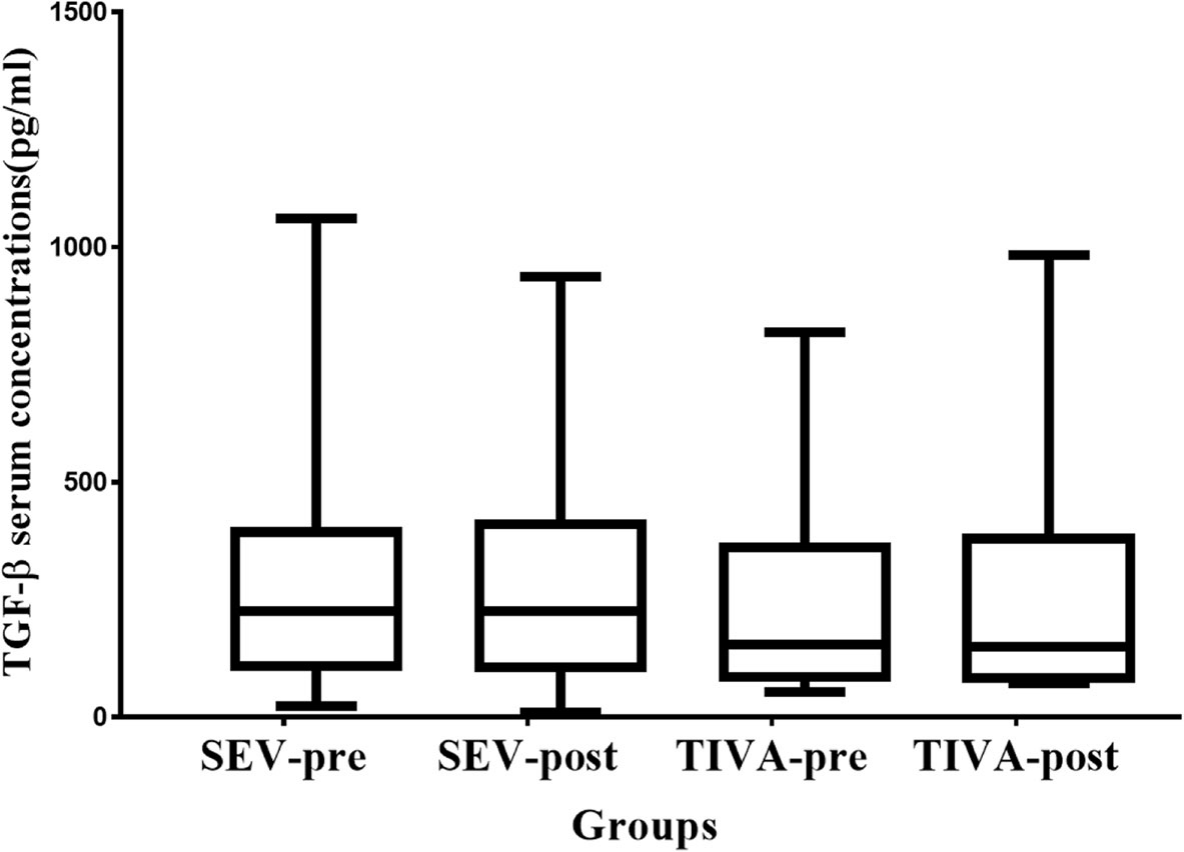
Median preoperative to postoperative changes of TGF-β concentrations of the patients with early stage cancer in both groups. There were13 and 14 patients with early stage cancer in the SEV and TIVA groups, respectively. SEV-pre preoperative values in the SEV group, SEV-post postoperative values in the SEV group, TIVA-pre preoperative values in the SEV group, TIVA-post postoperative values in the TIVA group. Horizontal line denotes median values, box borders refer to interquartile range, whiskers indicate range of values.

Serum concentrations of TGF-β after surgery didn’t show significant changes compared with the preoperative values in both groups. There were also no significant differences in the preoperative and postoperative TGF-β concentrations between the two groups (see Table 3). Interestingly, the median change of preoperative to postoperative TGF-β concentrations was − 14 (− 41–7) pg/ml among the patients with early stage (Tis-Stage I) tumors in the SEV group compared with 13 (− 9–55) pg/ml for those of the TIVA group (*P* = 0.038) (see Fig. 3).

### Changes of VEGF-C levels for MRM and BCS

In the SEV group, patients undergoing MRM had a significantly increased VEGF-C concentration of 197 (149–236) pg/ml after surgery compared with that of 104 (88–173) pg/ml before surgery (*p* < 0.001), while those receiving BCS had a postoperative VEGF-C concentration of 118(87–382) pg/ml similar to the preoperative value of 109(70–316) pg/ml (*P* = 0.734) (see Tables 4 and 5). In the TIVA group, VEGF-C concentrations were kept stable in the patients either receiving MRM (140[88–203] pg/ml vs. 149[110–280] pg/ml, *P* = 0.341) or BCS (127[59–213] pg/ml vs. 122[66–224] pg/ml, *P* = 0.752) (see Tables 4 and 5). Additionally, among the patients with MRM, the median preoperative to postoperative change of VEGF-C concentrations in the SEV group was 86 (30–116) pg/ml versus 22 (− 19–52) pg/ml in the TIVA group (*P* = 0.001).

**Table 4:** Comparisons of angiogenic factors concentrations (pg/ml) among the patients undergoing modified radical mastectomy between the two groups

	Group SEV (*n* = 23)	GroupTIVA (*n* = 24)	*P* -value
Preoperative values			
VEGF-C	104 (88–173)	140 (88–203)	0.285
TGF-β	183(114–272)	218(108–305)	0.666
Postoperative values			
VEGF-C	197 (149–236)	149(110–280)	0.193
TGF-β	184(135–299)	176(121–362)	0.708
Pre-post changes^a^			
VEGF-C	86(30–116)	22(−19–52)	0.001
TGF-β	12(−49–43)	10(− 42–51)	0.874

Values are median (25–75% interquartile range)

*Abbreviation*: *SEV* sevoflurane, *TIVA* total intravenous anesthesia, *VAS* visual analogue scale, *VEGF* vascular endothelial growth factor, *TGF* transforming growth factor

^a^Pre-post changes: postoperative values minus preoperative values

**Table 5:** Comparisons of angiogenic factors concentrations (pg/ml) among the patients undergoing breast conserving surgery between the two groups

	Group SEV (*n* = 17)	Group TIVA (*n* = 16)	*P*-value
Preoperative values			
VEGF-C	109(40–316)	127(59–213)	0.444
TGF-β	218(81–570)	183(94–294)	0.581
Postoperative values			
VEGF-C	118(87–383)	122(66–224)	0.488
TGF-β	226(106–544)	180(99–431)	0.743
Pre-post changes^a^			
VEGF-C	4(− 11–66)	7(− 4–64)	0.817
TGF-β	-4(−20–53)	13(−7–50)	0.383

Values are median (25–75% interquartile range)

*Abbreviation*: *SEV* sevoflurane, *TIVA* total intravenous anesthesia, *VEGF* vascular endothelial growth factor, *TGF* transforming growth factor

^a^Pre-post changes: postoperative values minus preoperative values

### RFS and OS rates at a two-year follow up

**Fig. 4:**
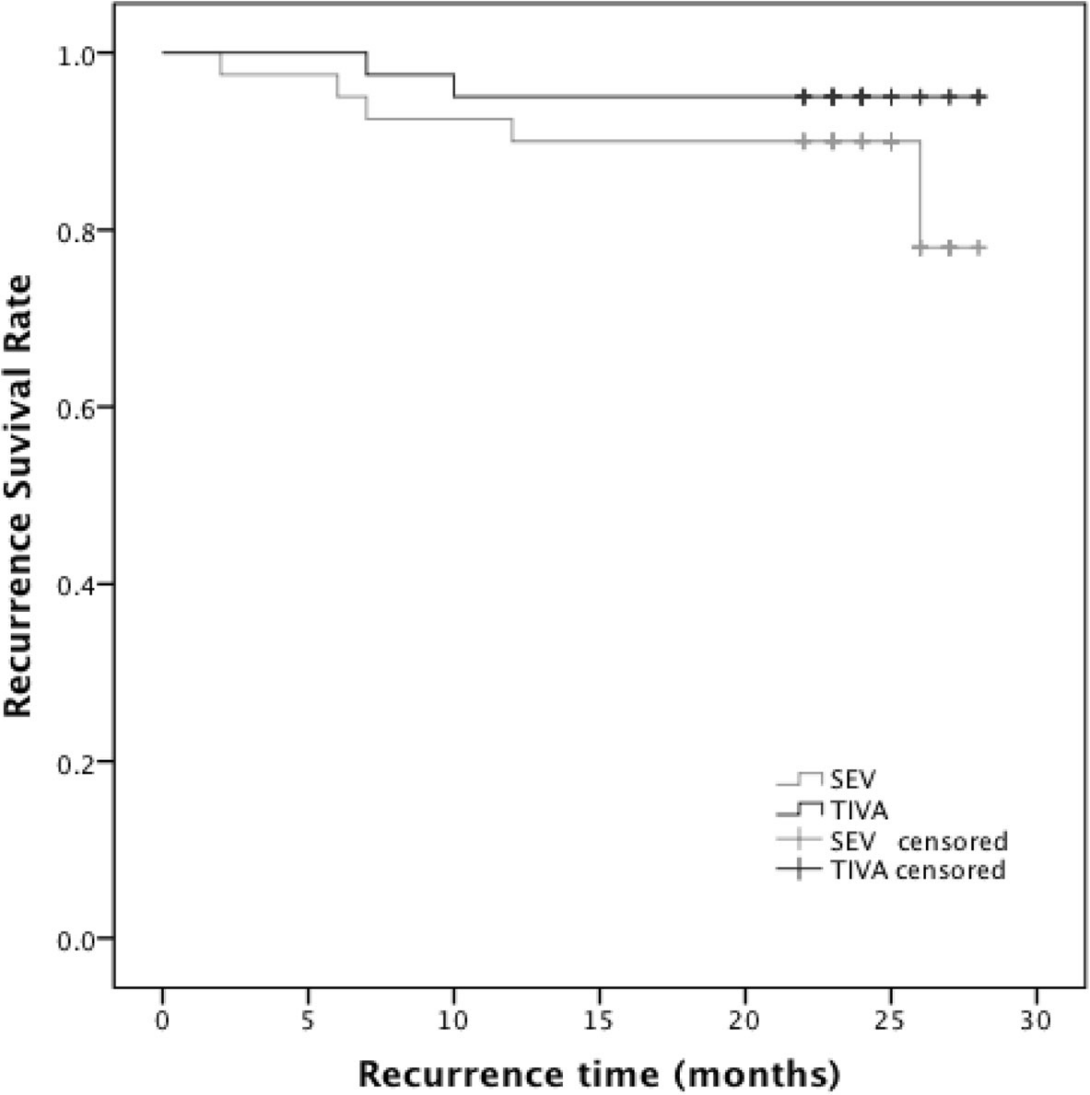
Kaplan-Meier recurrence-free survival estimated for 80 patients in both groups. Univariate analysis by log-rank test (*P* = 0.221)

Recurrences were documented in total 8 (10%) patients with 6 in the SEV and 2 in the TIVA groups during initial 28 months of follow up. Deaths were observed in total 2 patients with one in each group. The two-year RFS rates were 78% and 95% in the SEV and TIVA groups (*P* = 0.221), respectively, as shown in Fig. 4. The two-year OS rate was 97.5% in both groups.

## Discussion

Our study showed that propofol/remifentanil-based TIVA can effectively inhibit the increase of VEGF-C serum concentration induced by breast cancer surgery compared with sevoflurane-based inhalational anesthesia, especially for the patients receiving modified radical mastectomy. These two anesthetic methods had a comparable impact on the release of VEGF-C and TGF-β in the patients undergoing breast conserving cancer surgery. Furthermore, the short-term recurrence rates of breast cancer were similar between the two anesthetic groups.

It is plausible that surgery-induced activation of sympathetic nervous system and anesthetics related cell-mediated immunosuppression may promote angiogenesis through increasing the release of soluble factors such as VEGF and hypoxia-inducible factor1α [19, 20]. VEGF-C has been demonstrated to be overexpressed in the breast cancer [21] and increase metastatic potential of tumor cells by stimulating blood vessel growth [17]. TGF-β is also a potent angiogenic factor, but it can represent either tumorigenic or anti-tumor effect, depending on the presence and concentrations of other cytokines as well as its own concentration [2, 18].

A randomized controlled study suggested that propofol combined paravertebral block suppressed the increase in the postoperative concentrations of VEGF-C among the patients receiving breast cancer surgery compared with sevoflurane/morphine-based technique [2]. However, this result may be attributed to the combined effects of propofol, regional anesthesia and lack of opioids [22, 23]. Morphine at clinically relevant doses stimulated angiogenesis and caused an increase in breast cancer cells growth [24, 25]. Fentanyl and remifentanil decreased NK cell activity, but didn’t suppress immune resistance at low doses [10, 26, 27]. Furthermore, fentanyl has demonstrated anti-tumor property by inhibiting cancer cell migration and invasion [28].

In our study, propofol combined opioids rather than nerve block was used for breast cancer surgery because it was easily performed without nerve block associated adverse effects. Fentanyl was administrated in both groups at low doses of 2-4μg/kg, which were unlikely to impair antitumor immunity to a great extent [10]. Moreover, opioid- induced immunosuppression can be prevented by coadministration of cyclooxygase-2 (COX-2) inhibitor [29]. Propofol was shown to increase NK cell interferon-γ production and inhibit COX-2 and prostaglandin E2 function [30, 31], NASIDs was also used at the end of surgery in all patients, thereby possibly counteracting the negative impact of opioids on immunity [32]. However, Forget et al... suggested that NASIDs be given shortly before surgery to produce the antitumor effects [33]. In Looney et al’s study, propofol/paravertebral block produced lower VEGF-C concentration than sevoflurane technique [2], while out results showed the two groups had similar postoperative VEGF-C concentrations. This may be explained as that more patients in the TIVA group required postoperative fentanyl for pain relief due to remifentanil induced hyperalgesia [34] and after all, fentanyl could interfere with the anti-angiogenic effect of propofol.

In the present study, we also found propofol/remifentanil-based TIVA can effectively suppress the release of VEGF-C for both MRM and BCS. In contrast, sevoflurane technique can only reduce the expression of VEGF-C for BCS, but not for MRM. The contributory factors included that MRM caused greater stress response than BCS, hence more release of VEGF-C [35], and propofol-based TIVA can attenuate stress response more than inhalational anesthesia [36]. TGF-β concentrations remained relatively stable in all patients. Although among the patients with early stage (Tis-Stage I) tumors, the preoperative to postoperative change of TGF-β concentrations was lower in the SEV group compared with the TIVA group, the difference was too small to be clinically relevant.

We didn’t observe the beneficial effect of propofol/remifentanil-based TIVA on RFS during the two-year follow-up because the short-term RFS of breast cancer is relatively high, and a large sample size and a long-term follow-up would be required to detect a significant difference. In a prospective observational study, postoperative change in VEGF didn’t predict the disease-free survival in breast cancer over 8 years of follow-up [37].

There are some limitations worth noting in our study. First of all, propofol and fentanyl were used for anesthetic induction in both groups, especially, fentanyl was given in all patients to blunt intraoperative stress response and provide postoperative analgesia, which might serve as major confounders, making it difficult to differentiate the properties of two anesthetic methods on immune response. However, the effects of single-dose propofol used in the SEV group would have dissipated within 10 min or less as anesthesia was maintained with sevoflurane. And also, it was not substantially evidenced that small dose of fentanyl could make significant alterations on cancer related immunity. On the contrary, uses of opioids showed no clinically significant association with the recurrence of breast cancer in a large sample sized study [38]. More importantly, we aimed to compare the distinct packaged anesthetic techniques rather than attributing the results to the individual drug so as to present clinically relevant references. Moreover, the interactions of different anesthetics may further complicate the explanation for the results. Ultimately, whether our findings can be considered to indicate long-term prognosis is unknown.

## Conclusions

We found that for the breast cancer surgery, propofol/remifentanil-based TIVA can effectively inhibit the increases of VEGF-C concentrations after surgery compared with sevoflurane-based inhalational anesthesia, whereas these two anesthetic methods produced similar effects on the release of TGF-β. The short-term recurrence rate of breast cancer didn’t show a significant difference between the two anesthetic techniques. A large-sample sized, multicenter clinical trial with longer term follow-up should be conducted to clarify the roles of anesthetics on recurrence and metastasis of breast cancer.

## Abbreviations

ASA: American Society of Anesthesiology
BCS: Breast conserving surgery
BIS: Bispectral index
CMI: Cell-mediated immunity
COX-2: Cyclooxygase-2
ELISA: Enzyme-linked immunosorbent assay
ER: Estrogen receptor
HER2: Epidermal growth factor receptor2
MRM: Modified radical mastectomy
NK: Natural killer
NSAIDs: Nonsteroidal anti-inflammatory drugs
OS: Overall survival
PR: Progesterone receptor
RFS: Recurrence-free survival
SD: Standard deviation
SEV: Sevoflurane
TGF: Transforming growth factor
Tis: Tumor in situ
TIVA: Total intravenous anesthesia
VAS: Visual analogue scale
VEGF: Vascular endothelial growth factor

## References

[1] Siegel RL, Miller KD, Jemal A (2017). Cancer statistics, 2017. CA Cancer J Clin.

[2] Looney M, Doran P, Buggy DJ (2010). Effect of anesthetic technique on serum vascular endothelial growth factor C and transforming growth factor β in women undergoing anesthesia and surgery for breast cancer. Anesthesiology.

[3] Lee JH, Kang SH, Kim Y, Kim HA, Kim BS (2016). Effects of propofol-based total intravenous anesthesia on recurrence and overall survival in patients after modified radical mastectomy: a retrospective study. Korean J Anesthesiol.

[4] Zhang T, Fan Y, Liu K, Wang Y (2014). Effects of different general anesthetic techniques on immune responses in patients undergoing surgery for tongue cancer. Anaesth Intensive Care.

[5] Tavare AN, Perry NJ, Benzonana LL, Takata M, Ma D (2012). Cancer recurrence after surgery: direct and indirect effects of anesthetic agents. Int J Cancer.

[6] Ecimovic P, McHugh B, Murray D, Doran P, Buggy DJ (2013). Effects of sevoflurane on breast cancer cell function in vitro. Anticancer Res.

[7] Ecimovic P, Murray D, Doran P, Buggy DJ (2014). Propofol and bupivacaine in breast cancer cell function in vitro-role of the NET1 gene. Anticancer Res.

[8] Kushida A, Inada T, Shingu K (2007). Enhancement of antitumor immunity after propofol treatment in mice. Immunopharmacol Immunotoxicol.

[9] Ren XF, Li WZ, Meng FY, Lin CF (2010). Differential effects of propofol and isoflurane on the activation of T-helper cells in lung cancer patients. Anaestheisa.

[10] Yardeni IZ, Beilin B, Mayburd E, Alcalay Y, Bessler H (2008). Relationship between fentanyl dosage and immune function in the postoperative period. J Opioid Manag.

[11] Lin X, Wang YJ, Li Q, Hou YY, Hong MH, Cao YL (2009). Chronic high dose morphine treatment promotes SH-SY5Y cell apoptosis via c-Jun N-terminal kinase-mediated activation of mitochondria-dependent pathway. FEBS J.

[12] Juneja R (2014). Opioids and cancer recurrence. Curr Opin Support Palliat Care.

[13] Page GG, Ben-Eliyahu S, Yirmiya R, Liebeskind JC (1993). Morphine attenuates surgery-induced enhancement of metastatic colonization in rats. Pain.

[14] Page GG, McDonald JS, Ben-Eliyahu S (1998). Pre-operative versus postoperative administration of morphine: impact on the neuroendocrine, behavioural, and metastatic-enhancing effects of surgery. Br J Anaesth.

[15] Deegan CA, Murray D, Doran P, Ecimovic P, Moriarty DC, Buggy DJ (2009). Effect of anesthetic technique on oestrogen receptor-negative breast cancer cell function in vitro. Br J Anaesth.

[16] Gisterek I, Matkowski R, Lacko A, Sedlaczek P, Szewczyk K, Biecek P (2010). Serum VEGF A, C, D in human breast tumors. Pathol Oncol Res.

[17] Skobe M, Hawighorst T, Jackson DG, Prevo R, Janes L, Velasco P (2001). Induction of tumor lymphangiogenesis by VEGF-C promotes breast cancer metastasis. Nature Med.

[18] Dumont N, Arteaga CL (2000). Transforming growth factor-beta and breast cancer: tumor promoting effects of transforming growth factor-beta. Breast Cancer Res.

[19] Yang EV, Kim SJ, Donovan EL, Chen M, Gross AC, Webster Marketon JI (2009). Norepinephrine upregulates VEGF, IL-8, and IL-6 expression in human melanoma tumor cell lines: implications for stress related enhancement of tumor progression. Brain Behav Immun.

[20] Loop T, Dovi-Akue D, Frick M, Roesslein M, Egger L, Humar M (2005). Volatile anesthetics induce caspase-dependent, mitochondria mediated apoptosis in human T lymphocytes in vitro. Anesthesiology.

[21] Pinto MP, Badtke MM, Dudevoir ML, Harrell JC, Jacobsen BM, Horwitz KB (2010). Vascular endothelial growth factor secreted by activated stroma enhances angiogenesis and hormone-independent growth of estrogen receptor-positive breast cancer. Cancer Res.

[22] Exadaktylos AK, Buggy DJ, Moriarty DC, Mascha E, Sessler DI (2006). Can anesthetic technique for primary breast cancer surgery affect recurrence or metastasis?. Anesthesiology.

[23] Sacerdote P, Bianchi M, Gaspani L, Manfredi B, Maucione A, Terno G (2000). The effects of tramadol and morphine on immune responses and pain after surgery in cancer patients. Anesth Analg.

[24] Gupta K, Kshirsagar S, Chang L, Schwartz R, Law PY, Yee D (2002). Morphine stimulates angiogenesis by activating proangiogenic and survival-promoting signaling and promotes breast tumor growth. Cancer Res.

[25] Bimonte S, Barbieri A, Rea D, Palma G, Luciano A, Cuomo A (2015). Morphine promotes tumor angiogenesis and increases breast cancer progression. Biomed Res Int.

[26] Shavit Y, Ben-Eliyahu S, Zeidel A, Beilin B (2004). Effects of fentanyl on natural killer cell activity and on resistance to tumor metastasis in rats. Dose and timing study. Neuroimmunomodulation.

[27] Sacerdote P, Gaspani L, Rossoni G, Panerai AE, Bianchi M (2001). Effect of the opioid remifentanil on cellular immune response in the rat. Int Immunopharmocol.

[28] Li AX, Xin WQ, Ma CG (2015). Fentanyl inhibits the invasion and migration of colorectal cancer cells via inhibiting the negative regulation of Ets-1 on BANCR. Biochem Biophys Res Commun.

[29] Farooqui M, Li Y, Rogers T, Poonawala T, Griffin RJ, Song CW (2007). COX-2 inhibitor celecoxib prevents chronic morphine-induced promotion of angiogenesis, tumor growth, metastasis, and mortality, without compromising analgesia. Br J Cancer.

[30] Inada T, Kubo K, Shingu K (2010). Promotion of interferon-gamma production by natural killer cells via suppression of murine peritoneal macrophage prostaglandin E2 production using intravenous anesthetic propofol. Int Immunopharmacol.

[31] Inada T, Kubo K, Shingu K (2011). Possible link between cyclooxygenase-inhibiting and antitumor properties of propofol. J Anesth.

[32] Leahy KM, Ornverg RL, Wang Y, Zweifel BS, Koki AT, Masferrer JL (2002). Cyclooxygenase-2 inhibition by celecoxib reduces proliferation and induces apoptosis in angiogenic endothelial cells in vivo. Cancer Res.

[33] Forget P, Vandenhende J, Berliere M, Machiels JP, Nussbaum B, Legrand C (2010). Do intraoperative analgesics influence breast cancer recurrence after mastectomy? A retrospective analysis. Anesth Analg.

[34] Echevarría G, Elgueta F, Fierro C, Bugedo D, Faba G, Iñiguez-Cuadra R (2011). Nitrous oxide (N2O) reduces postoperative opioid-induced hyperalgesia after remifentanil-propofol anesthesia in humans. Br J Anaesth.

[35] Ben-Eliyahu S, Page GG, Yirmiya R, Shakhar G (1999). Evidence that stress and surgical interventions promote tumor development by suppressing natural killer cell activity. Int J Cancer.

[36] Ke JJ, Zhan J, Feng XB, Wu Y, Rao Y, Wang YL (2008). A comparison of the effect of total intravenous anesthesia with propofol and remifentanil and inhalational anesthesia with isoflurane on the release of pro- and anti-inflammatory cytokines in patients undergoing open cholecystectomy. Anaesth Intensive Care.

[37] Rocca A, Cancello G, Bagnardi V, Sandri MT, Torrisi R, Zorzino L (2009). Perioperative serum VEGF and extracellular domains of EGFR and HER2 in early breast cancer. Anticancer Res.

[38] Cronin-Fenton DP, Heide-Jørgensen U, Ahern TP, Lash TL, Christiansen PM, Ejlertsen B (2015). Opioids and breast cancer recurrence: a Danish population-based cohort study. Cancer.

